# Boiling local heat transfer enhancement in minichannels using nanofluids

**DOI:** 10.1186/1556-276X-8-130

**Published:** 2013-03-18

**Authors:** Ali Ahmad Chehade, Hasna Louahlia Gualous, Stephane Le Masson, Farouk Fardoun, Anthony Besq

**Affiliations:** 1 Université de Caen Basse Normandie, LUSAC, 120 rue de l’exode, Saint Lo, 50000, France; 2 France Telecom, Orange Labs, 2 av Pierre Marzin, 22/300, Lannion, France; 3 Department of Industrial Engineering and Maintenance, Lebanese University, Saida, 1600, Lebanon

**Keywords:** Minichannels, Nanofluid, Convective boiling

## Abstract

This paper reports an experimental study on nanofluid convective boiling heat transfer in parallel rectangular minichannels of 800 μm hydraulic diameter. Experiments are conducted with pure water and silver nanoparticles suspended in water base fluid. Two small volume fractions of silver nanoparticles suspended in water are tested: 0.000237% and 0.000475%. The experimental results show that the local heat transfer coefficient, local heat flux, and local wall temperature are affected by silver nanoparticle concentration in water base fluid. In addition, different correlations established for boiling flow heat transfer in minichannels or macrochannels are evaluated. It is found that the correlation of Kandlikar and Balasubramanian is the closest to the water boiling heat transfer results. The boiling local heat transfer enhancement by adding silver nanoparticles in base fluid is not uniform along the channel flow. Better performances and highest effect of nanoparticle concentration on the heat transfer are obtained at the minichannels entrance.

## Review

### Introduction

The rapid improvement in the microelectronic devices is accompanied by a high increase in the heat generation, which would decrease its efficiency and lifetime. Nanofluid flow boiling in microchannels and minichannels came up to be a novel solution to withstand high heat fluxes with low working mass flow rates and more uniform temperature. Thus, the combination of nanofluid and small channel’s dimensions in heat exchangers constitutes an innovating method providing effectiveness, compactness, low thermal resistance, and, simultaneously, environmental protection by the reduction of working fluid inventory.

Several studies were carried out to better understand the boiling phenomena in microchannels with different working fluids [1,2]. Bowers and Mudawar [3] conducted experiments in circular minichannels and microchannels heat sinks by using R-113 as a working fluid. They found that minichannels and microchannels in heat exchangers are capable of achieving heat fluxes in excess of 200 W/cm^2^. Moreover, Qu and Mudawar [4] investigated convective boiling heat transfer, flow patterns, and pressure drop of water in parallel microchannels. They showed that the flow pattern was strongly affected by the heat flux and it is difficult to withstand bubbly flow regimes using water as working fluid due to its high surface tension and large contact angle. Liu and Garimella [5] conducted experiments on boiling heat transfer of deionized water in copper microchannels. They found that Shah correlation [6] predicts well the heat transfer coefficient in the subcooled boiling regimes. Chen and Garimella [7] investigated physical characteristics of boiling FC-77 flow in parallel silicon minichannels. They studied bubbly and sluggish flow pattern at low heat flux and thin annular and churn flows at high heat flux using three different mass fluxes. Fang et al. [8] conducted a comparative study of existing correlations for flow boiling heat transfer in microchannels. They collected 1158 data points of flow boiling heat transfer of R134a in minichannels and reviewed 18 flow boiling heat transfer correlations. They found that no correlation has satisfactory accuracy and that more efforts should be made to develop better correlations for boiling in minichannels.

In addition, the recent development of nanotechnology materiel led to intensify the heat transfer coefficient in microscale devices by using suspended metallic nanoparticles in conventional working fluids. Most studies published in the literature on nanofluids heat transfer have reported that using nanoparticles with average sizes below than 100 nm in traditional working fluids increases the thermal conductivity of fluids and enhances heat transfer coefficient [9,10]. Mohammed et al. [11] reported that there are few studies on convective heat transfer compared to those on nanofluid properties because forced convective flows are affected by nanofluids thermal properties in addition to the Reynolds and Prandtl numbers. Consequently, there are many experimental studies, which focused on nanofluids thermal conductivities since it is the most important parameter to enhance convective heat transfer. Among many experimental methods reported in the literature to measure the nanofluids thermal conductivity, the transient hot wire method has been used extensively. Various correlations and models were proposed for the calculation of the thermal conductivity of nanofluids [12,13].

In contrast, nanofluids in microchannels have received little attention. Few numerical and experimental studies have been conducted on convection nanofluid heat transfer in microchannels for single phase and boiling flows [14,15]. Various sizes and types of nanoparticles have been tested such as Al_2_O_3_, CuO, diamond, SiO2, Ag, and TiO2 s. These studies have revealed that the heat transfer performance and pressure drop increase with increasing nanoparticle volume concentration in base fluid and decrease with increasing nanoparticle size.

Regarding boiling heat transfer using nanofluids as working fluids, it can be seen that there are several published researches on pool boiling [16,17]. However, few studies on convective boiling heat transfer of nanofluid in microchannels or minichannels have been conducted in the past 3 years [18-20]. Boudouh et al. [21] conducted experiments on heat transfer of nanofluid with three different volume fractions of nanoparticles in the base fluid 0.00056%, 0.0011%, and 0.0056%. They showed that the local heat flux, local vapor quality, and local heat transfer coefficient increase with copper nanoparticle volume fraction. Henderson et al. [22] found that the heat transfer coefficients of the R134a/POE/CuO nanofluid could be increased by 52% and 76% for volume fractions of 0.04% and 0.08% respectively. Kim et al. [23] studied Al_2_O_3_-water nanofluid at low volume concentration and observed an enhancement of the boiling critical heat flux up to 70% at nanoparticle concentrations lower than 0.01%. They attributed this enhancement to the nanoparticle deposition on the heat exchanger surface. On the other hand, Lee and Mudawar [24] tested two volume fractions of Al_2_O_3_-water nanofluid (1% and 2%) with diameter of 36 nm. They noted that the boiling of nanofluid could fail since large clusters are formed near the channel exit due to localized evaporation once boiling was started. More recently, Xu and Xu [25] investigated flow boiling heat transfer in a single microchannel using 40 nm Al_2_O_3_ nanoparticles with low volume fraction (0.2%). They showed that nanofluids stabilize the boiling flow and inhibit the dry patch development between the heater surface and vapor phase. They also observed an enhancement of the heat transfer using nanofluid without particle deposition on the heater surface.

From this literature review, it is clear that there are only limited studies on nanofluid boiling heat transfer in microchannels with low volume concentration. Most of the studies are focused on pool boiling and single-phase heat transfer in microchannels. Additionally, the encouraging results of a few research works on boiling heat transfer in microchannels at very low nanoparticle volume fractions show the possibility of employing boiling nanofluid in micro heat sinks. Therefore, more efforts must be made in this field to improve effectiveness in engineering designs and applications.

The objective of this study is to investigate the boiling thermal performance of water-based silver nanoparticles in rectangular minichannels. Experiments were conducted with pure water and nanofluids having low nanoparticle concentrations. The results of local heat transfer coefficients for both water and nanofluids were compared under steady state. Effects of the suspended silver nanoparticles in water on the local surface temperature, local heat flux, and local heat transfer coefficient are also analyzed.

### Experimental setup

#### Flow loop

**Figure 1:**
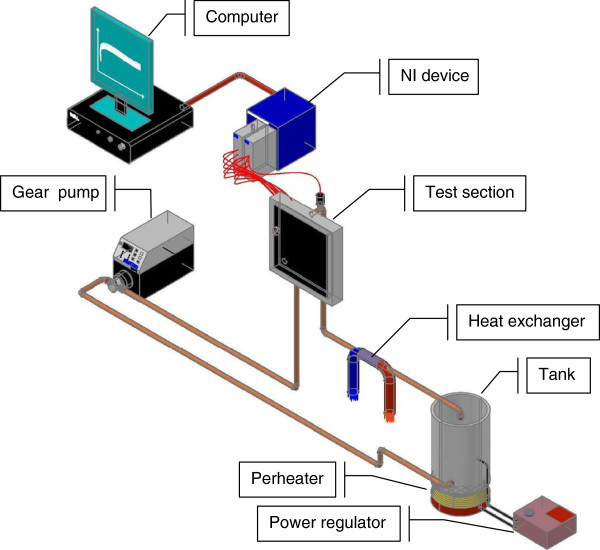
A schematic diagram of the experimental apparatus.

Figure 1 shows a schematic diagram of the test setup that has been built to conduct experiments for boiling local heat transfer in the minichannels. The test setup consists of fluid loop with working fluid reservoir and a preheater, variable speed gear pump, test section, heat exchanger, power regulator, thermocouples, computer, and acquisition data devices. The working fluid temperature at the vented reservoir is controlled at a desired temperature by a preheater that consists of resistance, temperature regulator, and a K-type sensor. In addition, the reservoir volume is large enough to take back all the fluid when the facility is shut down. The magnetic MCP-Z standard drive gear pump circulates the working fluid to the test section from the vented reservoir. Water exiting the test section is cooled via a heat exchanger before reaching the reservoir. The 75 μm K-type thermocouples are used to measure the inner wall temperature of the minichannels. The whole test rig is fully automated through a computer using the National Instruments devices (National Instruments Corp., Austin, TX, USA).

#### Test section

**Figure 2:**
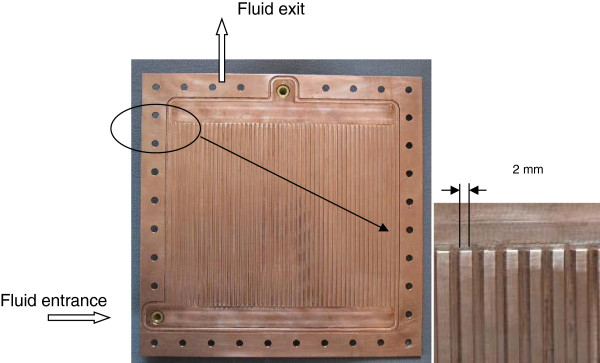
Top view of the test section with 50 minichannels.

**Figure 3:**
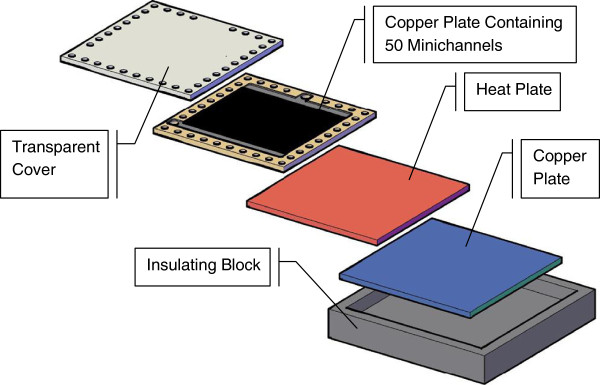
Detailed test model assembly.

Figure 2 presents the top view of the test section consisting of a 220 × 220 × 10 mm^3^ copper block. Fifty parallel rectangular channels are machined on the block's upper side. Each channel has a rectangular cross section (2,000 μm width and 500 μm height) and a length of 160 mm. The distance between the center lines of the two adjacent channels is 4 mm. Figure 3 shows the test model assembly. The flow channels are formed by covering the top side of the copper plate with a polycarbonate plate of 220 × 220 × 4 mm^3^ which is also used as an insulator and a transparent cover in order to visualize the boiling flow patterns. The parallel minichannels are heated by a rectangular silicone heating panel of 200 × 200 × 4 mm^3^ placed at the copper plate bottom side. The heater system is coated with a second copper plate 200 × 200 × 4 mm^3^. These two copper blocks are screwed into place so that they made good contact with the heater source. Precautions were taken to achieve uniform distribution of heat flux at the upper surface of the heat source. The heating panel was fed with a direct current power supply that has 400 W total powers. The input voltage and current are controlled by a power supply device and measured with an accuracy of 1%. As shown in Figure 3, thermal insulating layers (30-mm thick) of PTFE with thermal conductivity 0.3 W/mK are placed on all faces of the test section except the top side in order to minimize the heat losses which are estimated to be lower than 7%.

#### Instrumentation

**Figure 4:**
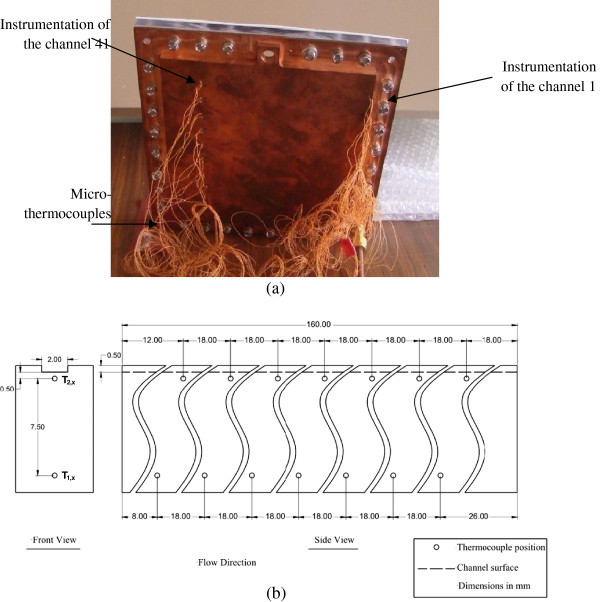
**Bottom of the test section and location of thermocouples inside copper plate wall.** (**a**) Bottom views of the test section showing the implemented thermocouples and (**b**) location of thermocouples inside copper plate wall for the first channel.

To understand the physical phenomena, experimental setup and local instrumentation have been developed and experiments were conducted. The inner wall temperature of the minichannels is measured using K-type microthermocouples of 75 μm diameter. Microthermocouples are inserted in drillings on the back side of the copper plate as shown in Figure 4a. They were soldered using a high-conductivity material along the walls of the first and 41th minichannels. The first minichannel is located at 2 mm from the edge of the test section, near the entry of the working fluid. The channel 41 is located at 160 mm far from the edge of the test section. At the first channel 7, microthermocouples were implemented at 0.5 mm below the wetted surface at 12, 30, 48, 66, 103, 121, and 139 mm from the channel inlet. In addition, seven microthermocouples were implemented at 8 mm below the wetted surface at 8, 26, 44, 63, 98, 116, and 134 mm from the channel inlet (as shown in Figure 4b). Regarding channel 41, nine thermocouples were implemented at 0.5 mm below the wetted surface at 10, 28, 46, 62, 83, 101, 119, 137, 154 mm from the channel inlet. In addition, seven microthermocouples were implemented at 8 mm below the wetted surface at 14, 50, 36, 68, 86, 104, 123, and 159 mm from the channel inlet. A high-speed camera is installed in front of the test section to visually record the flow evolution. Data acquisition is entirely automated using the Labview data acquisition system (National Instruments Corp., Austin, TX, USA).

#### Experimental procedure, data reduction, and uncertainties

For all tests, the heat exchange surface was oriented vertically. The liquid in the tank was first preheated to the required temperature. The liquid flow rate was adjusted with a regulating valve at the desired value. All temperatures were recorded during time. The total power supplied to the heater source was set at the maximum value. When the boiling phenomenon had occurred and the temperatures have reached almost a steady state, the values of the liquid flow rate or the heat flux of the power source were varied and the same procedure was repeated. For each fixed experimental condition, the test section was heated and the temperatures were monitored continually. Experiments were performed with deionized water and silver-water nanofluids.

Experimental results presented in this paper were treated only in the steady state when the wall temperatures become approximately constant with time. The temperatures fluctuation is about ±0.1°C. The local heat transfer coefficient of each axial location along the channel length is given as follows:

(1) $$h_{x}=\frac{q_{\mathrm{channel},\;x}}{T_{s,x}-T_{f}}$$

where *q*_channel, *x*_ is the local heat flux estimated by taking into account the local heat loss, *T*_s,*x*_ is the local surface temperature, *T*_f_ is the fluid bulk mean temperature, and *x* is the axial coordinate parallel to the flow's direction.

The local heat flux is calculated depending on Fourier’s law:

(2) $$q_{\mathrm{channel},\;x}=\lambda_{w}\frac{T_{1,x}-T_{2,x}}{\mathrm{\Delta y}}$$

where *λ*_w_(=389 W/mK) is the thermal conductivity of the copper wall, *T*_1,*x*_ and *T*_2,*x*_ are the temperatures measured inside the copper plate, Δ*y* is the space between thermocouples locations inside the wall (see Figure 4b).

The vapor quality is defined as the ratio of the local vapor mass flow rate to the total mass flow rate $\left(\chi_{v,x}={\dot{m}}_{v,x}/\left({\dot{m}}_{v,x}+{\dot{m}}_{l,x}\right)\right)$. Applying the energy balance equation between the inlet and the outlet of each subsection yields

(3) $$\begin{array}{l} \chi_{v,x}=\chi_{v,x-\mathrm{\Delta x}}+\frac{1}{h_{\mathrm{fg}}}\left[\frac{q_{\mathrm{channel},x}+q_{\mathrm{channel},x-\mathrm{\Delta x}}}{2}\right)\\ \;\left(\times ,\frac{\mathrm{\Delta x}\;W_{\text{channel}}}{\dot{m}},-,C_{\mathrm{pl}},\left(T_{\mathrm{sat}}-T_{f}\right)\right] \end{array}$$

where *q*_channel*,x*_ is the local heat flux along the flow direction, *h*_*fg*_ is the heat of vaporization, *W*_channel_ is the channel width, *T*_sat_ is the working fluid saturation temperature, *T*_f_ is the working fluid inlet temperature, *C*_pl_ is the liquid working fluid specific heat capacity, and $\dot{m}$ is the single channel mass flow rate determined from the assumption that the total mass flow rate is uniformly distributed in the minichannels,

(4) $$\dot{m}=G\frac{H_{\mathrm{channel}}W_{\mathrm{channel}\;}}{N_{\mathrm{channel}}}$$

where *G* is the total mass flux measured during experiments, *H*_channel_ is the channel height, *W*_channel_ is the channel width, and *N*_channel_ is the number of channels.

A Denver Instrument flow meter (Bohemia, NY, USA) is used to measure the mass flow rate of the working fluid with an uncertainty of 1.3%. Furthermore, microthermocouples calibration is carried out by comparing the temperatures measured by each microthermocouple to those measured by a high-precision sensor probe (±0.03°C). The uncertainties in heat flux, heat transfer coefficient, vapor quality, and mass flux (Equations 1, 2, 3, and 4) were evaluated using the method of Kline and McClintock [26]. For example, the uncertainty of the heat flux was evaluated by the following:

(5) $$\frac{\mathrm{\Delta q}}{q}={\left[{\left(\frac{\Delta \lambda }{\lambda }\right)}^{2}+{\left(\frac{\Delta T_{1}}{T_{2}-T_{1}}\right)}^{2}+{\left(\frac{\Delta T_{2}}{T_{2}-T_{1}}\right)}^{2}+{\left(\frac{\Delta_{\mathrm{\Delta y}}}{\mathrm{\Delta y}}\right)}^{2}\right]}^{\frac{1}{2}}$$

where *q* is the heat flux along the flow direction, *λ* the thermal conductivity of the copper plate, *T* is the temperature measured inside the copper plate for different levels, Δ*y* is the space between thermocouples locations inside the copper plate. Table 1 shows the uncertainties for different parameters involved in the measurements.

**Table 1:** Uncertainties for different parameters involved in the experimental tests

**Parameter**	**Uncertainty**
Temperature, *T* (°C)	±0.1°C
Mass flow rate, $\dot{m}$ (kg/s)	±1.3%
Mass flux, *G* (kg/m^2^s)	±1.35%
Position of thermocouples, *y* (m)	±0.1 mm
Power input, (W)	1%
Heat flux, *q* (W/m^2^)	8%
Heat transfer coefficient, *h* (W/m^2^k)	±12%

## Results and discussion

Experiments are performed in parallel rectangular minichannels using pure water and silver-water nanofluid with two small volume fractions (0.000237% and 0.000475%) as working fluids in a compact heat exchanger. A comparison between proposed correlations in the literature and experimental data is carried out initially to verify the present measurements and then to evaluate correlations defined for flow boiling heat transfer in minichannel or macrochannel. Experiments are conducted with various values of mass flux and heat flux.

### Water boiling heat transfer in minichannels: measurement results and predictions

#### Transient state: temperature measurements and instability

**Figure 5:**
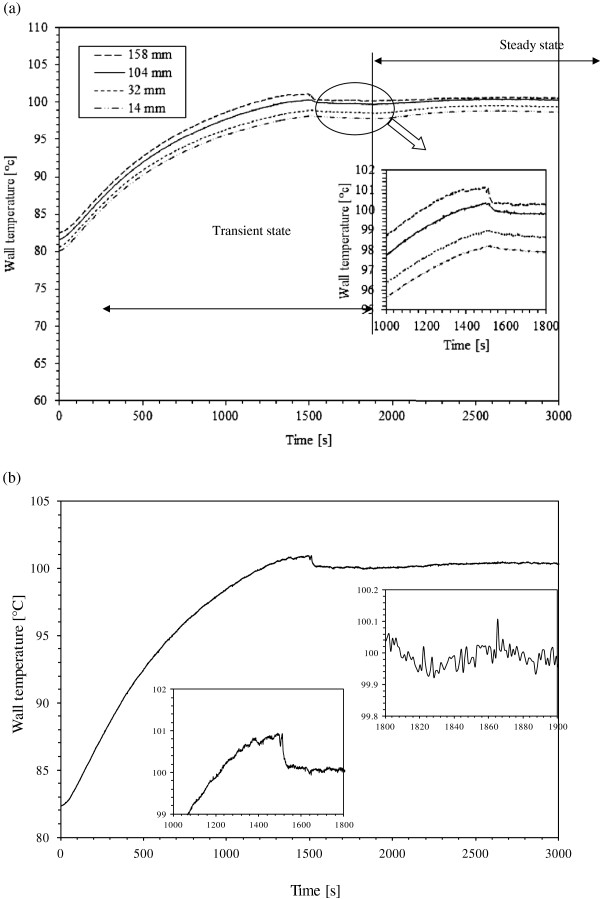
**Evolution of the wall temperature.** (**a**) Measurements by various thermocouples along the flow direction for 0.5 mm depth and (**b**) example of wall temperature fluctuations.

For each operating conditions, wall temperatures are measured at different axial locations of the minichannels. Figure 5a shows an example of four transient temperatures profiles measured at 0.5 mm below the heat exchange surface along the flow direction. The experiment is conducted for 60°C inlet water temperature, 266 kg/m^2^s mass flux and 200 W supplied power to the heated plate. The figure shows that the wall temperatures increase regularly during transient state with some fluctuations (Figure 5b) until a limit is reached then decrease at the start of the nucleate boiling to reach steady values. Figure 5b shows an example of the wall temperature fluctuations in the steady state zone caused by the hydrodynamic instabilities of the bubbles and liquid flows. In a previous work, it was revealed that various types of hydrodynamic instabilities may exist in boiling flow and boiling flow has a destabilizing effect on the two-phase flow. In this study, experimental data show that bubbles generated on the heated surface move to the channel exit and coalesce with other bubbles to feed the high void fraction. Flow oscillation in the minichannels may be attributed to the difference between the vapor and the liquid densities. Instability in boiling flow can reduce the critical heat flux due to the flow oscillation that tends to increase the bubble velocity along the channel. Previously, Qu and Mudawar [4] showed that pressure drop oscillation is undesirable for the performance of a two-phase microchannel heat sink.

#### Steady state: temperature and heat transfer coefficient measurements

**Figure 6:**
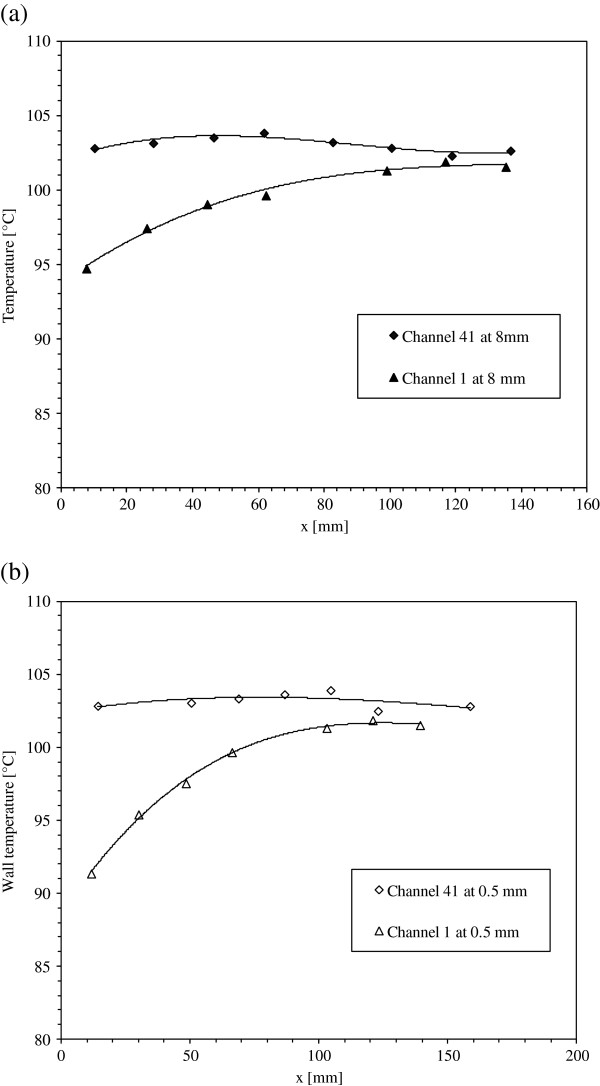
**Wall temperature measurements of channels 1 and 41 with 348 kg/m**^**2**^**s pure water mass flux at (a) 8-mm depth and (b) 0.5-mm depth.**

**Figure 7:**
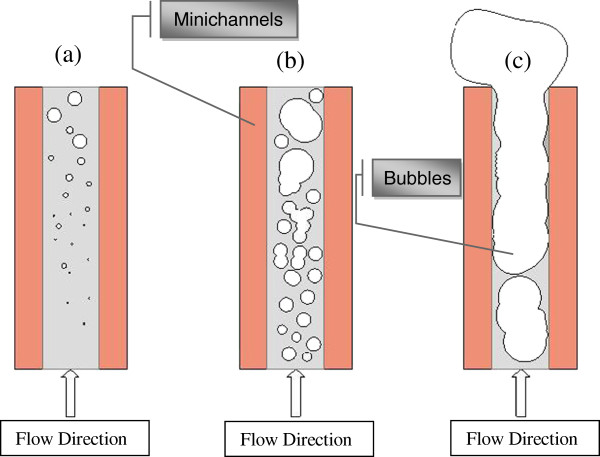
**Boiling flow pattern at different locations along the flow direction.** (**a**) *x* ≤ 80 mm, (**b**) 60 mm ≤ *x* ≤ 110 mm, and (**c**) 100 mm ≤ *x* ≤ 160 mm.

Figure 6a,b shows an example of the wall temperature measured at 0.5 and 8 mm below the heat exchange surface for the channels 1 and 41 for 348 kg/m^2^s pure water mass flux. The total power supplied to the heated plate is 200 W. It is shown that for both channels, the wall temperatures increase along the flow direction and attain a horizontal asymptote at the downstream flow. For the channel 41, all the measurement locations show a very low wall temperature variation (approximately isotherm) along the channel, leading a uniform distribution of the big bubbles along the channel. Wall temperature distribution along the channel is related to the boiling flow structure where it increases with the size of the bubbles in the channel. Moreover, three zones along the flow direction are observed as shown in Figure 7. The first zone (Figure 7a) is at the channel entrance where the nucleate boiling begins and a small number of isolated bubbles move just after their apparition along the liquid flow. The first zone length may be reduced by decreasing the fluid mass flow rate or by increasing the heat flux. Bubbles leaving the first zone combine with bubbles formed in the second zone (Figure 7b) to form bigger bubbles occupying the middle part of the channel. The increase of the bubble size decreases the contact of water with the heat exchange surface and increases the wall temperature. At the upstream flow, a third zone is observed (Figure 7c), where the temperature and void fraction attain their maximum values causing probably a partial dry regions near the channels’ outlet. As a result, wall temperature and local vapor quality increase along the flow direction.

**Figure 8:**
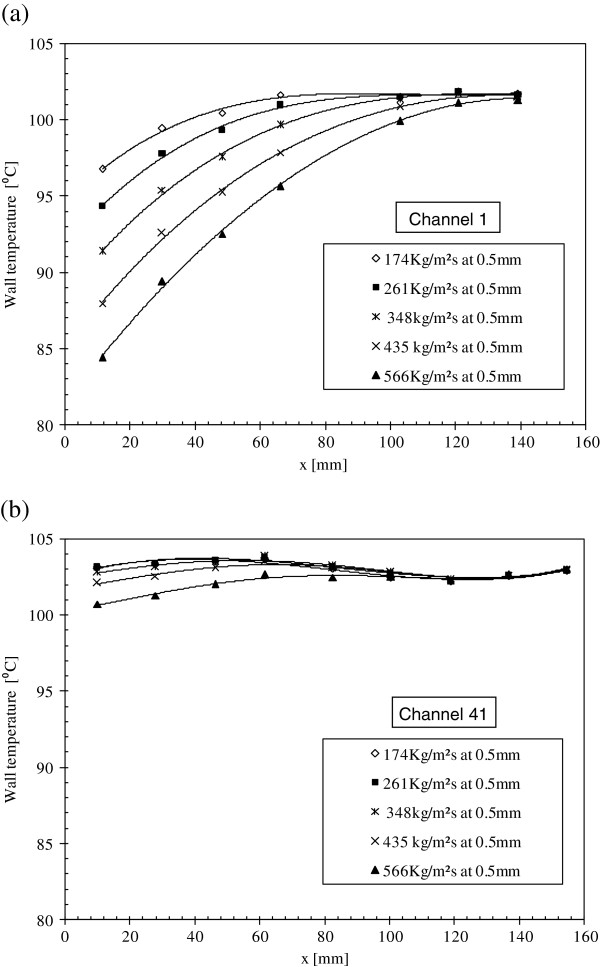
Wall temperature measurements for different pure water mass fluxes, (a) channel 1 and (b) channel 41.

The effect of the water mass flux on the wall temperature evolution is presented in Figure 8a,b. The profiles of wall temperatures measured at the first and 41th channel along the flow direction using microthermocouples located at 0.5 mm below the heat exchange surface are shown. The pure water mass fluxes for these profiles are 174, 261, 348, 435, and 566 kg/m^2^s, where the total power supplied to the heated plate is 200 W. Figure 8a shows a strong dependence of the wall temperature on the liquid's mass flux. As the liquid's mass flux increases, the wall temperature decreases and vice versa. Moreover, all the curves attain a horizontal asymptote at the end of the channel length, i.e., at the maximum local vapor quality. In addition, it can be noticed that the zone's length where the wall temperature becomes asymptotic increases as liquid's mass flux decreases and vice versa. In fact, for the same heat flux, the decrease of the mass flow rate increases both the local void fraction and the local wall temperature. For channel 41, the effect of the mass flux on the wall temperature is quite low in comparison with the wall temperature obtained for the first channel since the void fraction in this channel is higher along the flow direction and that can be seen in Figure 8b.

**Figure 9:**
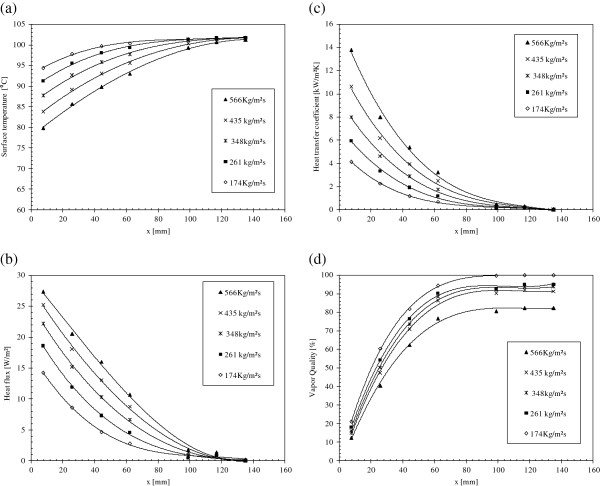
**Heat transfer parameters for different mass fluxes.** (**a**) Local heat transfer coefficient, (**b**) local heat flux, (**c**) surface temperature, and (**d**) vapor quality.

Afterward, the heat transfer parameters can be calculated depending on the previous Equations 1, 2, and 3. Figure 9a,b,c,d shows the local surface temperature, local heat flux, local heat transfer coefficient, and the local vapor quality, respectively, along the flow direction for different pure water mass fluxes. Experimental data show a strong dependence of the local heat transfer coefficient and local heat flux on the liquid's mass flux and on the *x* location. They possess almost the same shapes with decreasing local heat transfer coefficient and local heat flux, with the increase of *x* and decrease of liquid's mass flux. For the same mass flux, the surface temperature at the downstream flow is smaller and the local heat transfer coefficient is greater than those at the upstream flow. At the channel's inlet, the nucleate boiling dominates causing a high heat transfer coefficient and low surface temperature. But while moving toward upstream flow, the vapor covers the major part of the flow outlet and prevents the contact between liquid flow and the channels' surface causing a partial dry out and blockage mechanisms which, in turn, causes a decrease in the local heat transfer coefficient and an increase in the surface temperature. As shown in Figure 9d, the local vapor quality increases along the channel’s length and with smaller water mass fluxes.

#### Comparison of experimental data with the existing correlations for flow boiling heat transfer

In order to validate the experimental procedure, experimental results obtained in the present work for boiling water in minichannels are compared to predictions of various correlations from literature. These existing correlations are proposed for convective boiling heat transfer in microchannels and macrochannels (Table 2). Of these predictive correlations, those for boiling flow in the rectangular minichannels defined by Warrier et al. [27], Kandlikar and Balasubramanian [28], Sun and Mishima [29] and Bertsch et al. [30] are employed. On the other hand, Fang et al. [8] compared experimental data for convective boiling of R113 in minichannels with the predictions from 18 correlations defined for flow boiling heat transfer. They found that the best predictions of the average boiling heat transfer coefficient are found with a mean absolute relative deviation of 36% by the correlations of Lazarek and Black [31] and Gungor and Winterton [32], which are developed for convective boiling in macrochannels. Predictions from these two correlations are also compared to the experimental data. The correlation of Kew and Cornewell [33] established for boiling heat transfer in macrotubes is also evaluated in this paper. Yan and Lin [34] investigated experiments on evaporation heat transfer in multi-port circular tube with an inner diameter of 2 mm. They proposed an equation for heat transfer similar to the Kandlikar [2] correlation, including three non-dimensional numbers: the boiling number, the liquid Froude number, and the convection number (Table 3). Cooper’s correlation [35] that is developed and widely used for nucleate pool boiling heat transfer is recommended by Harirchian et al. [1] to predict flow boiling heat transfer in microchannels. However, Harirchian et al. [1] found that the Cooper’s correlation predicts their experimental results with 27% as mean absolute percentage error. Liu and Witerton [36] used Cooper’s correlation and introduced an enhancement factor due to the forced convective heat transfer mechanism caused by bubbles generated in the flow. Bertsch et al. [30] developed a generalized correlation for flow boiling heat transfer in channels with hydraulic diameters ranging from 0.16 to 2.92 mm. The proposed correlation by Bertsch et al. [30] predicts these measurements with a mean absolute error less than 30%.

**Table 2:** Correlations for boiling flow heat transfer coefficient

**Reference**	**Fluid composition**	**Description**			**Correlation**
		**Geometry**	**Comment**	**Parameter range**	
Warrier et al. [27]	FC-84	Small rectangular parallel channels of *D*_h_ = 0.75mm	Single-phase forced convection and subcooled and saturated nucleate boiling	3 <*x* <55%	$h_{\mathrm{tp}}=h_{\mathrm{sp}}\left(1+6{\mathrm{Bo}}^{\frac{1}{16}}-5.3\left(1-855\mathrm{Bo}\right)\chi_{v,x}^{0.65}\right)\;\left(6\right)$ $h_{\mathrm{sp}}=0.023Re_{l}^{0.8}Pr_{l}^{0.4}\lambda_{l}/D_{h}\;\left(7\right)$
Kandlikar and Balasubramanian [28]	Water, refrigerants, and cryogenic fluids	Minichannels and microchannels	Flow boiling	*x* <0.7 ~ 0.8	$\mathrm{Co}<0.65,h_{\mathrm{tp}}=h_{\mathrm{sp}}\left[1.136{\mathrm{Co}}^{-0.9}{\left(25Fr_{\mathrm{lo}}\right)}^{c}+667.2{\mathrm{Bo}}_{\mathrm{lo}}^{0.7}\right]\;\left(8\right)$ $\mathrm{Co}>0.65,h_{\mathrm{tp}}=h_{\mathrm{sp}}\left[0.6683{\mathrm{Co}}^{-0.2}{\left(25Fr_{\mathrm{lo}}\right)}^{c}+1058{\mathrm{Bo}}_{\mathrm{lo}}^{0.7}\right]\;\left(9\right)$ *h*_sp_ is calculated Equation 7
Sun and Mishima [29]	Water, refrigerants (R11, R12, R123, R134a, R141b, R22, R404a, R407c, R410a) and CO2	Minichannel diameters from 0.21 to 6.05 mm	Flow boiling laminar flow region	*Re*_*L*_ < 2,000 and *Re*_*G*_ < 2,000	$h_{\mathrm{tp}}=\frac{6Re_{\mathrm{lo}}^{1.05}{\mathrm{Bo}}^{0.54}\lambda_{l}}{{\mathrm{We}}_{l}^{0.191}{\left(\rho_{l}/\rho_{g}\right)}^{0.142}D_{h}}\;\left(10\right)$
Bertsch et al. [30]	Hydraulic diameters ranging from 0.16 to 2.92 mm	Minichannels	Flow boiling and vapor quality	0 to 1	$h_{\mathrm{tp}}=\left(1-\chi_{v,x}\right)h_{\mathrm{nb}}+\left[1+80\left(\chi_{v,x}^{2}-\chi_{v,x}^{6}\right)e^{-0.6{\mathrm{Co}}_{f}}\right]h_{\mathrm{sp}}\;\left(11\right)$ *h*_nb_ is calculated by Cooper [35]: $h_{\mathrm{nb}}=55P_{R}^{0.12-0.087\ln\xi }{\left(-0.4343\ln P_{R}\right)}^{-0.55}M^{-0.5}q^{0.67}\;\left(12\right)$ *h*_sp_ = *χ*_v,*x*_*h*_sp,go_ + (1 − *χ*_v,*x*_)*h*_sp,lo_ (**13**) $h_{\mathrm{sp},\mathrm{ko}}=\left[3.66+\frac{0.0668Re_{\mathrm{ko}}Pr_{k}D_{h}/L}{1+0.04{\left(Re_{\mathrm{ko}}Pr_{k}D_{h}/L\right)}^{2/3}}\right]\frac{\lambda }{D_{h}}\;\left(14\right)$ ${\mathrm{Co}}_{f}=\sqrt{\frac{\sigma }{g\left(\rho_{l}-\rho_{g}\right)D_{h}^{2}}}\;\left(15\right)$
			Temperature	−194°C to 97°C	
			Heat flux	4–1,150 kW/m^2^	
			Mass flux	20–3,000 kg/m^2^s	
Lazarek and Black [31]	R113	Macrochannels 3.15 mm inner diameter tube	Saturated flow boiling	-	$Nu_{x}=30Re_{\mathrm{lo}}^{0.857}{\mathrm{Bo}}^{0.714}\;\left(16\right)\;$
Gungor and Winterton [32]	Water and refrigerants (R-11, R-12, R-22, R-113, and R-114)	Horizontal and vertical flows in tubes and annuli *D* = 3 to 32 mm	Saturated and subcooled boiling flow	0.008 <*p*_sat_ < 203 bar; 12 <*G* < 61.518 kg/m^2^s; 0 <*x* < 173%; 1 <*q* < 91.534 kW/m^2^	*h*_tp_ = (*SS*_2_ + *FF*_2_)*h*_sp_ (17) *h*_sp_ is calculated Equation 6 *S* = 1 + 3, 000Bo^0.86^ (18) $F=1.12{\left(\frac{\chi_{v,x}}{1-\chi_{v,x}}\right)}^{0.75}{\left(\frac{\rho_{l}}{\rho_{g}}\right)}^{0.41}\;\left(19\right)$ S2=Frlo(0.1-2Fr)loifhorizontalwithFrlo<0.051otherwise20 $$F_{2}=\left\{\begin{array}{l} Fr_{\mathrm{lo}}^{\left(0.5\right)}\;\mathrm{if}\;\mathrm{horizontal}\;\mathrm{with}\;Fr_{\mathrm{lo}}<0.05\\ 1\;o\mathrm{therwise} \end{array}\right)\;\left(21\right)$$
Liu and Witerton [36]	Water, refrigerants and ethylene glycol	Vertical and horizontal tubes, and annuli	Subcooled and saturated flow boiling	-	$h_{\mathrm{tp}}=\sqrt{{\left(Fh_{\mathrm{lo}}\right)}^{2}+{\left(Sh_{\mathrm{nb}}\right)}^{2}}\;\left(22\right)\;$ *h*_nb_ is calculated by Cooper [35] (Equation 11) $F=0.35\left[1+\chi_{v,x}\frac{\mu_{l}C_{p,l}}{\lambda_{l}}\left(\frac{\rho_{l}}{\rho_{v}}-1\right)\right]\;\left(23\right)$ $S=\left[1+0.055F^{0.5}Re_{\mathrm{lo}}^{0.16}\right]\;\left(24\right)$
Kew and Cornwell [33]	R141b	Single tubes of 1.39–3.69 mm inner diameter	Nucleate boiling, confined bubble boiling, convective boiling, partial dry out	-	$h_{\mathrm{tp}}=30Re_{\mathrm{lo}}^{0.857}{\mathrm{Bo}}^{0.714}\frac{\lambda_{l}}{D_{h}}{\left(\frac{1}{1-\chi_{v,x}}\right)}^{0.143}\;\left(25\right)$
Yan and Lin [34]	R134a	28 parallel tubes 2 mm	Convective boiling	*G* = 50 to 200 kg/m^2^s; *q* = 0.5 to 2 W/cm^2^	$h_{\mathrm{tp}}=\left(C_{1}{\mathrm{Co}}^{C_{2}}+C_{3}{\mathrm{Bo}}^{C_{4}}Fr_{\mathrm{lo}}\right){\left(1-\chi_{v,m}\right)}^{0.8}h_{l}\;\left(26\right)$ *h*_l_ = 4.364*λ*_l_/*D*_h_ (**27**) Cm=Cm,1ReloCm,2TRCm,328 The best fitting values for the constants *C*_*m*,1_, C_*m*,2_, and *C*_*m*,3_ are listed in Table 3

**Table 3:** **Values of the constants in Yan and Lin**[34]**correlation**

**Average**	**Co *>*0.5**			**0.15<Co ≤ 0.5**			**Co ≤ 0.15**		
	***C***_***m*,1**_	***C***_***m*,2**_	***C***_***m*,3**_	***C***_***m*,1**_	***C***_***m*,2**_	***C***_***m*,3**_	***C***_***m*,1**_	***C***_***m*,2**_	***C***_***m*,3**_
1	933.6	0.07575	26.19	47.3	0.3784	14.67	356600	−0.6043	18.59
2	−0.2	0	0	2612.8	0	37.27	1409.1	−0.5506	16.303
3	21700	0.5731	34.98	100150	0	24.371	12.651	0.3257	10.118
4	14.84	−0.0224	13.22	3.99	−0.1937	4.794	0.15	0	0

**Figure 10:**
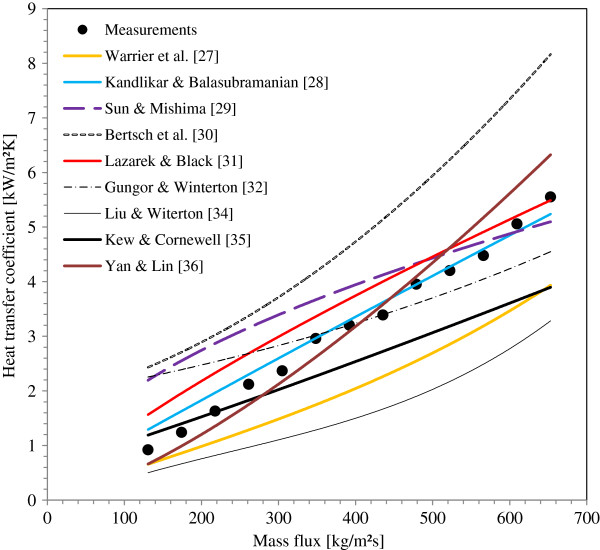
Comparison between the predicted and the measured average heat transfer coefficients for different mass fluxes.

Comparisons between the present experimental results to the predictions from these correlations are illustrated in Figure 10. Kandlikar and Balasubramanian [28] correlation best predicts the heat transfer coefficients measured in the present work. Predictions of heat transfer from the correlations of Lazarek and Black [31] and Yan and Lin [34] are very satisfactory for all the tested mass fluxes. The maximum deviation is about 29% for mass flux ranging from 260 to 650 kg/m^2^s. However, Sun and Mashima [29] correlation gives the best predictions for high mass flux (>450 kg/m^2^s) with an average deviation about 13% from the measurements and over predicts measurements for low mass fluxes. Also, correlation of Bertsch et al. [30] highly over predicts the experimental results for all the range of mass flux tested in this study and the correlations of Liu and Witerton [36] and Warrier et al. [27] under predict them. Correlations of Gungore and Winterton [32] and Kew and Cornewell [33] have the same trend to over predict the heat transfer coefficient at low mass flux and to under predict them at high mass flux. Table 4 presents the percentage dispersion of the proposed correlations relative to the experimental average heat transfer coefficient measured at different water mass fluxes.

**Table 4:** Standard deviation of the various correlations with respect to experimental results

***G *value (kg/m**^**2**^**)**	**Measurement results**	**Warrier et al.**[27]**(%)**	**Kandlikar and Balasubramanian**[28]**(%)**	**Sun and Mishima**[29]**(%)**	**Bertsch et al.**[30]**(%)**	**Lazarek and Black**[31]**(%)**	**Gungor and Winterton**[32]**(%)**	**Liu and Witerton**[36]**(%)**	**Kew and Cornwell**[33]**(%)**	**Yan and Lin**[34]**(%)**
130.59	0.92	−27.89	41.6	133.99	166.33	65.87	188.31	−32.68	16.22	−19.64
174.12	1.24	−31.37	30.34	97.03	130.45	60.27	93.15	−60.02	33.67	−8.55
217.65	1.63	−34.92	20.25	80.65	100.28	45.09	67.84	−43.69	−1.22	−6.23
261.18	2.12	−38.41	10.32	48.89	44.37	25.75	16.35	−58.02	−18.09	−26.22
304.71	2.37	−36.85	10.14	50.32	53.31	29.29	8.49	−56.62	−20.13	−22.64
348.24	2.96	−40.13	0.84	25.01	30.2	11.31	−10.39	−59.7	−25.52	−25.17
391.77	3.2	−38.46	1.54	28.33	60.69	14.79	2.17	−47.7	−17.36	−5.16
435.3	3.39	−33.23	6.6	26.66	69.24	27.36	4.72	−42.28	−14.41	11.49
478.83	3.95	−35.52	−0.32	13.33	60.17	3.62	−3.11	−43.35	−20.11	14.45
522.36	4.2	−31.93	2.24	6.52	38.53	17.09	−19.72	−52.51	−26.04	4.7
565.89	4.48	−29.01	2.21	3.02	47.22	−0.97	−16.04	−47.65	−25.47	22.78
609.42	5.06	−29.69	−0.56	−5.43	41.32	5.61	−19.94	−48.04	−29.81	25.42
652.95	5.55	−29.21	−7.08	−10.67	53.45	12.48	5.53	−36.92	−28.05	29.41

### Nanofluids boiling heat transfer in minichannels

**Figure 11:**
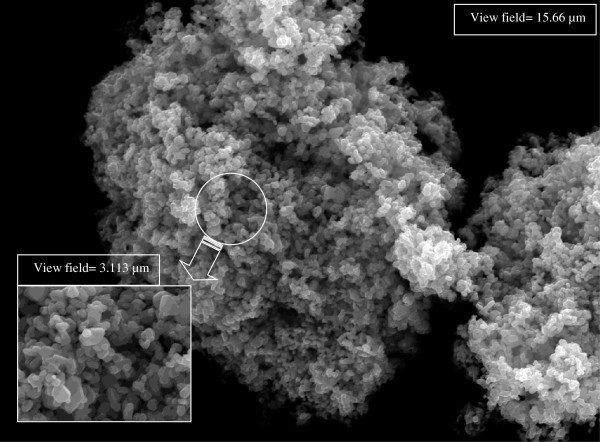
Silver nanoparticles with an average diameter of 35 nm.

Nanofluid is prepared and used as a working fluid for the boiling apparatus. Silver nanoparticles with 35 nm diameter are dispersed in the deionized water base solution. Figure 11 shows the silver nanoparticles photo used in this work. An ultrasonic vibrator is used for about one day to insure the best dispersion of the silver nanoparticles in the deionized water. Moreover, nanofluid is directly tested after preparation since the nanoparticles would coagulate together to form big particles. Experiments are conducted to measure nanofluid boiling heat transfer with two nanoparticle concentrations of 50 mg/L and 25 mg/L corresponding to 0.000475% and 0.000237% nanoparticle volume fractions, respectively, which are quite low compared to those used for boiling in minichannels by previous research works. No dispersant fluid is added during the nanofluid preparation. For each concentration, nanofluid mass flux is varied at the inlet of the minichannels, and the test section is cleaned after each experiment using deionized water.

#### Effect of silver nanoparticles on the local heat transfer

Among the various equations defined in the literature to compute the physical properties of nanofluid, the most used correlations have been retained in this work to estimate nanofluid properties. The following equations are used to calculate the nanofluid thermal conductivity, dynamic viscosity, density, and specific heat respectively [24,37]:

(29) $$\lambda_{\mathrm{eff}}=\left[\frac{\lambda_{p}+\left(n-1\right)\lambda_{\mathrm{bf}}-\phi \left(n-1\right)\left(\lambda_{\mathrm{bf}}-\lambda_{p}\right)}{\lambda_{p}+\left(n-1\right)\lambda_{\mathrm{bf}}+\phi \left(\lambda_{\mathrm{bf}}-\lambda_{p}\right)}\right]\lambda_{\mathrm{bf}},$$

where *n = 3* for spherical nanoparticle,

(30) $$\frac{\mu_{\mathrm{nf}}}{\mu_{\mathrm{bf}}}=1+2.5\phi ,$$

(31) $$\rho_{\mathrm{nf}}=\phi \rho_{p}\left(1-\phi \right)\rho_{\mathrm{bf}},$$

(32) $$C_{p,\mathrm{nf}}=\phi C_{p,p}\left(1-\phi \right)C_{p,\mathrm{bf}},$$

where *λ* is the thermal conductivity, *ϕ* is the nanoparticle volume fraction, *μ*_b_ is the viscosity of the base fluid, *ρ* is the density, and *C*_p_ is the specific heat capacity.

Table 5 shows the physical properties of water base fluid and silver-water nanofluids with different nanoparticle volume fractions.

**Table 5:** Pure water and nanofluid properties at 100 kPa and 60°C

	**Water**	**Silver nanoparticles**	**Silver nanofluid (*C *= 25 mg/L)**	**Silver nanofluid (*C *= 50 mg/L)**
Effective thermal conductivity *λ* (mw/mK)	603	429	603.427	603.856
Density *ρ* (kg/m^3^)	996	10490	998.25	1000.51
Dynamic viscosity *μ* (kg/ms)	7.977 × 10^−4^	-	0.000798	0.0008
Specific heat, *C*_p_ (J/kgK)	4,182	233	4181.064	4180.124

**Figure 12:**
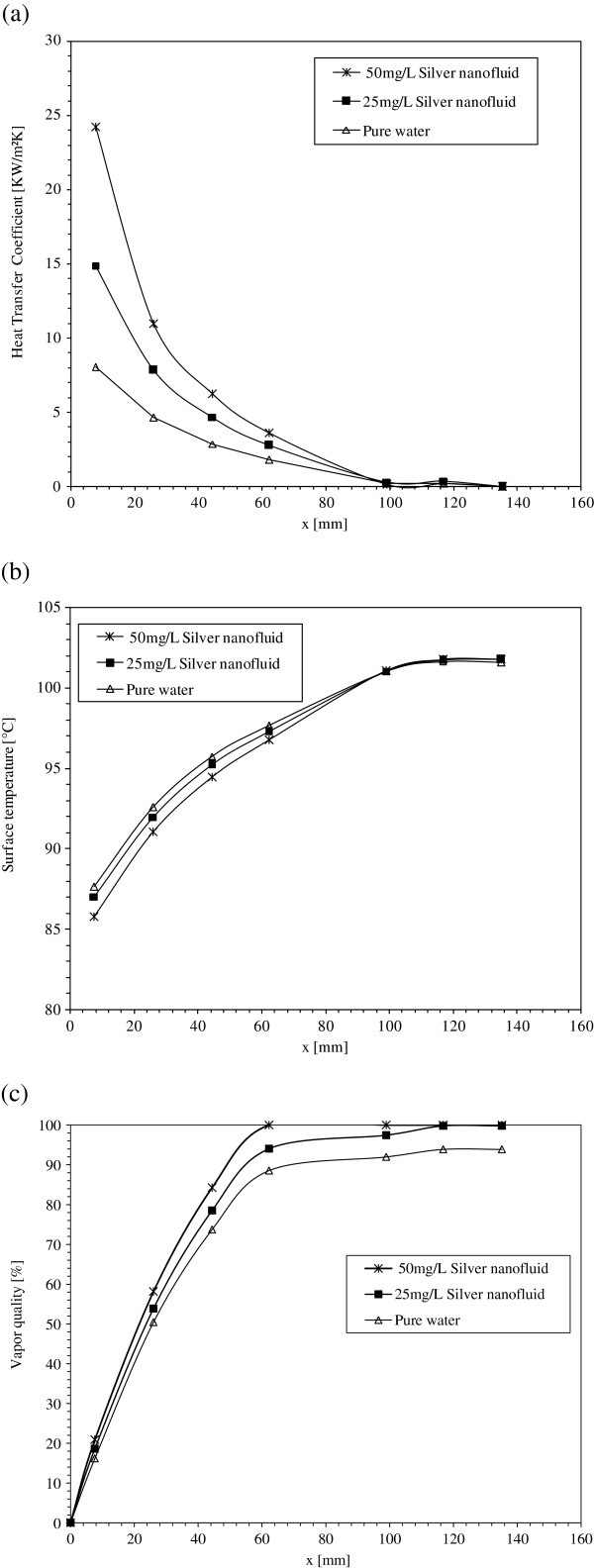
**Heat transfer parameters for pure water, 25 and 50mg/L concentration silver nanofluids along the minichannel length.** (**a**) Local heat transfer coefficient, (**b**) surface temperature, and (**c**) vapor quality.

Figure 12a,b,c presents distributions of the local heat transfer coefficient, local surface temperature, and local vapor quality respectively along the minichannel length. Each figure compares the experimental data obtained for boiling flow of pure water to those of nanofluids with 25 and 50 mg/L silver concentrations. The inlet working fluid mass flux is 348 kg/m^2^s with an input heat power of 200 W. Figure 12a reveals that the local heat transfer coefficient increases with silver nanoparticles suspended in deionized water. It could be noticed that the enhancement in the local heat transfer coefficient is very appreciable near the channel entrance. Figure 12b demonstrates that the surface temperature decreases by increasing silver nanoparticle concentration in the water base fluid due to the increase in the heat transfer and the cooling of the heat exchange surface. This is confirmed by Figure 12c showing that nanofluids give higher vapor quality than pure water. Therefore, the increase of the silver nanoparticle concentration increases the local heat transfer coefficient and the vapor quantity in the boiling flow, and reduces the surface temperature.

#### Effect of silver nanoparticles on the average heat transfer

**Figure 13:**
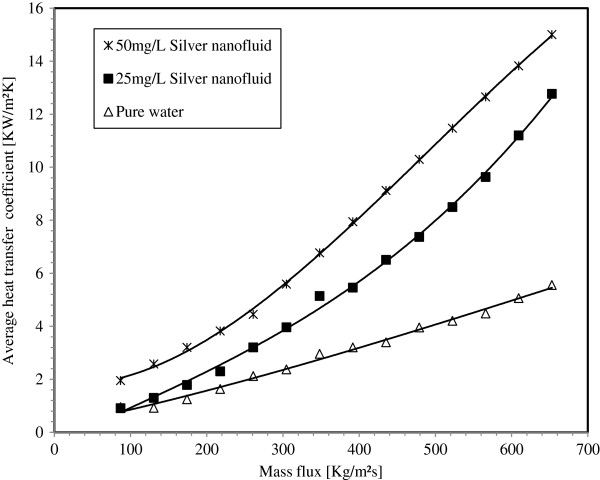
Average heat transfer coefficient in function of the mass flux for an input power of 200 W.

**Figure 14:**
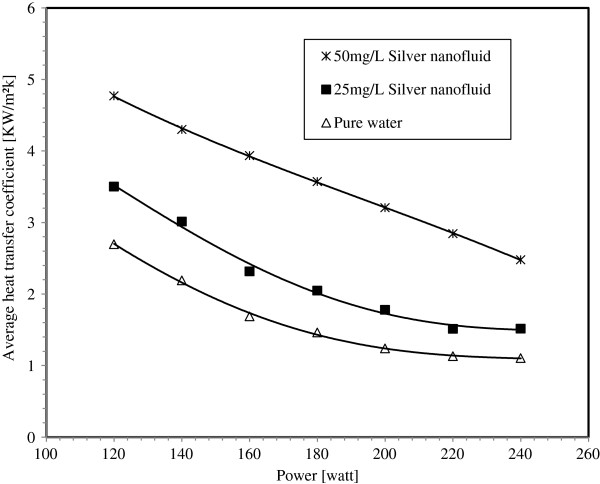
Variation of the average heat transfer coefficient with heater's power.

Two experimental conditions are conducted for each silver nanoparticle concentration in water base fluid and pure water. In the first one, the input power is settled at 200 W and the mass flux is varied from 87 to 653 kg/m^2^s. In the second, the mass flux is settled at 174 kg/m^2^s and the input power is varied from 120 to 240 W. Figure 13 compares the average heat transfer coefficients of pure water, 25 mg/L and 50 mg/L silver concentration nanofluid under the first experiment conditions. For the same mass flux, the average heat transfer coefficient is larger for nanofluids than that of pure water and it is increased with nanoparticle suspension. The maximum enhancement of the average heat transfer coefficient is about 132% for 25 mg/L and 162% for 50 mg/L. Figure 14 illustrates experimental data obtained under the second experiment conditions. It can be seen that the average heat transfer coefficient for pure water and silver-water nanofluids increases by decreasing the input power. For the whole input power range, the heat transfer coefficients have almost the same trends for boiling silver-water nanofluids and water. For each fixed power input value, increasing the silver nanoparticle concentration will increase the average heat transfer coefficient. Accordingly, for an input power ranging from 120 to 240 W, the enhancement of the average heat transfer coefficient for nanofluids relative to pure water is about 30% to 38% for 25 mg/L and 56% to 77% for 50 mg/L silver concentrations, respectively.

In general, heat transfer enhancement using nanofluid has being investigated by many researchers, and several mechanisms leading to this enhancement are presented, such as nanoparticle interactions with bubbles [38], nanoparticle porous deposition on the surface [20], reduction of the thermal boundary layer thickness due to nonuniform distribution of the thermal conductivity and viscosity [39], increase in the viscosity and decrease in the thermal capacity [40], chaotic movement, and dispersion and fluctuation of nanoparticles [41]. Also, as explained by Wen and Ding [37], nanofluid improves the convection heat transfer coefficient because of nanoparticle rotation and the associated microconvection. However, Xu and Xu [25] attributed enhancement of nanofluid heat transfer to the increase of the thin liquid film evaporation. It has been found by several researchers [42,43] that bubble diameters increase using nanofluids boiling, but the nucleation site density decreases. In the boiling field, further studies on bubble dynamics and on the heat transfer of nanofluid microlayer evaporation will provide valuable information about the physical mechanisms controlling heat transfer enhancement when adding nanoparticles to the base fluid.

## Conclusions

This article presents experimental results of convective boiling local heat transfer in rectangular minichannels using nanofluids as the working fluids. It shows that both local heat transfer coefficient and local heat flux are affected equally by the concentration of nanoparticles suspended in water base fluid and the structure of the boiling flow in minichannels. The main concluding points of the investigated experiments in this study are the following:

1. Among all correlations employed in the present work, only Kandlikar and Balasubramanian [28] correlation best predicts the heat transfer coefficients for convective boiling in minichannels. Those of Lazarek and Black [31] and Yan and Lin [34] established for macrochannels give satisfactory estimation of boiling heat transfer coefficient with the standard deviation of 29%. However, Sun and Mashima [29] correlation gives the best predictions with standard deviation of 13% for high mass flux only, but it over predicts measurements for low mass fluxes.

2. Adding silver nanoparticles in the water base fluid enhances the boiling local heat transfer coefficient, local heat flux, and local vapor quality, and reduces the surface temperature compared to pure water.

3. The boiling local heat transfer enhancement with silver-water nanofluid is highest in the minichannel entrance region where the vapor quality is low, and it decreases along the flow direction. The enhancement of the local heat transfer coefficient can reach 86% and 200% for 25 mg/L and 50 mg/L silver concentrations in water-based fluid, respectively.

4. At high vapor quality, the presence of silver nanoparticles in water base fluid has no effect on the boiling local heat transfer coefficient, which decreases dramatically.

5. Suspension of silver metallic nanoparticles in water base fluid at very low concentration can significantly increase the heat transfer performance of the miniature systems. The maximum enhancement of the average heat transfer coefficient is about 132% and 162% for 25 mg/L and 50 mg/L silver concentrations nanofluid respectively. Using nanofluids, at low nanoparticle concentrations, in minichannels or microchannels can be considered as the potential revolution in heat transfer enhancement processes for many industries' applications.

## Abbreviations

Bo: boiling number, *q*_channel,*x*_ / (*Gh*_*fg*_); Co: convection number, (1/*χ*_v,*x*_ − 1)^0.8^(*ρ*_g_ / *ρ*_l_)^0.5^; C: concentration; Cp: specific heat capacity (J/kgK); Dh: hydraulic diameter (m) 2*H*_channel_*W*_channel_ / (*H*_channel_ + *W*_channel_); e: channel thickness (m); F: forced convection enhancement factor; Fr: Froude number, *G*^2^/(*ρ*^2^g*D*_h_); g: gravity (m/s^2^); G: mass flux (kg/m^2^s); h: heat transfer coefficient (W/m^2^K); hfg: latent heat of vaporization (J/kg); Hchannel: channel height (m); L: channel length (m);$\overset{\cdot }{m}$: mass flow rate (kg/s); M: molar mass (kg/kmol); Nchannel: number of channels; Nu: Nusselt number; PR: reduced pressure, *P*/*P*_crit_; Pr: Prandtl number; q: heat flux (W/m^2^); Re: Reynolds number, *GD*_h_/*μ*; S: nucleate boiling suppression factor; T: temperature (K); TR: reduced temperature, *T*/*T*_crit_; Wchannel: channel width (m); We: Weber number, *G*^2^*D*_h_/(*σρ*_l_); χ: vapor quality; x: axial coordinate (m); y: axial coordinate (m). Greek letters: Δ, increment, standard deviation; λ: thermal conductivity (W/m K); ξ: channel surface roughness (μm); μ: dynamic viscosity (kg/ms); ρ: density (kg/m^3^); σ: surface tension (N/m); φ: volume fraction. Subscripted letters: bf, base fluid; crit: critical point; eff: effective; g: gas; go: gas only; f: fluid; k: index, gas or liquid: l, liquid; lo: liquid only; m: average; nb: nucleate boiling; nf: nanofluid; p: solid nanoparticles; s: surface; sat: saturation; sp: single phase; tp: two phase; v: vapor; w: wall; x: local value.

## Competing interests

The authors declare that they have no competing interests.

## Authors’ contributions

AC, HLG and SL jointly did the planning of the experiments, analysis of the data, and writing the manuscript. They did the synthesis, characterization, and the measurements. FF helped on the redaction of the manuscript and analysis of the data. AB participated in the characterization of the nanoparticles size and in the preparation of nanofluids. All authors read and approved the final manuscript.
